# Improving Access to Emergency Healthcare for Inpatients of Mental Health Hospitals: A Service Evaluation

**DOI:** 10.1192/bjo.2026.11590

**Published:** 2026-06-30

**Authors:** Poppy Shelton, Rebecca Anne McMillan, Matthew Roughley, Asho Oommen, Elizabeth Sampson

**Affiliations:** 1 East London Foundation Trust, London, United Kingdom; 2 Barts Health NHS foundation trust, London, United Kingdom; 3 London's Air Ambulance, London, United Kingdom; 4 Academic Centre for Healthy Ageing, London, United Kingdom; 5 Queen Mary University of London, London, United Kingdom

## Abstract

**Aims:**

To assess the impact of a pilot project, which aimed to improve access to emergency healthcare for inpatients of mental health hospitals, by collecting qualitative feedback from clinicians and patients.

**Background:**

Patients admitted to mental health inpatient wards often have significant chronic health comorbidity and a reduced life expectancy by 15-20 years with challenges in the provision of physical health care in these settings. A pilot project ran between December 2024 – May 2025 whereby the Psychiatric duty doctor discussed patients with acute physical health needs with the Remote Access Emergency Coordination Hub (REACH), rather than calling 999. The patient was either conveyed to the ED, the Physician Response Unit (PRU) was dispatched, or they were managed with alternative follow up arranged by the REACH. REACH received 62 phone referrals in 6 months and of these only 14 were ultimately conveyed to ED.

**Methods:**

Feedback was gathered during the 6 month pilot project by disseminating questionnaires to the REACH, PRU and mental health clinicians. General comments were organised into themes. Appropriate patients who had contact with the pathway were contacted via telephone, 3 patients were able to provide feedback.

**Results:**

Key themes that emerged were; high quality appropriate referrals, good clear handover, good approach to security and safety, clearly communicated plans from PRU, integration between physical and mental health care, importance of access to ED senior clinician, increasing scope of service and improving patient care.

Patient recollection of been seen by PRU was limited due to them being acutely unwell at the time.

**Conclusion:**

This is an important service, integrating physical and mental health care to improve outcomes for a vulnerable group of patients. The process of referring to REACH was clear and the plans well communicated. The majority of the emergency department team felt the referrals from the psychiatry duty doctor were appropriate, of good quality and received an adequate handover. It is important for PRU staff to be met by a staff member from the mental health unit to ensure safety and security. Poor patient recollection of the service highlights the medical and psychiatric complexity of the patients PRU/REACH were contacted about.

